# Adaption of pregnancy anxiety questionnaire–revised for all pregnant women regardless of parity: PRAQ-R2

**DOI:** 10.1007/s00737-015-0531-2

**Published:** 2015-05-14

**Authors:** A. C. Huizink, M. J. Delforterie, N. M. Scheinin, M. Tolvanen, L. Karlsson, H. Karlsson

**Affiliations:** Department of Developmental Psychology & EMGO+ Institute for Health and Care Research, VU University Amsterdam, Van der Boechorststraat 1, 1081 BT Amsterdam, Netherlands; FinnBrain Birth Cohort Study, Turku Brain and Mind Center, Institute of Clinical Medicine, University of Turku, Turku, Finland; Department of Child Psychiatry, Turku University Hospital, Turku, Finland; Department of Psychiatry, Turku University Hospital, Turku, Finland

**Keywords:** Pregnancy, Anxiety, Measurement invariance, Parity, Pregnancy-related anxiety questionnaire–revised

## Abstract

The 10-item Pregnancy-Related Anxiety Questionnaire–Revised (PRAQ-R) is a widely used instrument to assess and identify pregnancy-specific anxiety in nulliparous women. It has good psychometric values and predictive validity for birth and childhood outcomes. Nonetheless, the PRAQ-R is not designed for use in parous women, as particularly one item of the questionnaire is not relevant for women who gave birth before. We tested the factorial and scalar invariance of a modified PRAQ-R2 across nulliparous and parous women with an adapted item to fit both groups of pregnant women. A longitudinal study among 1144 pregnant women (*n* = 608 nulliparous and *n* = 536 parous) with two repeated measures of the PRAQ-R2 was used to test for measurement invariance of the instrument. Results show metric and scalar invariance, indicating that the PRAQ-R2 measures similar constructs on the same scale for all pregnant women at two different times during pregnancy. We conclude that the PRAQ-R2 can be used, compared, or combined in a sample of nulliparous and parous women.

## Introduction

Feelings of anxiety across pregnancy are relatively common, with about 10–15 % of all pregnant women experiencing some level of anxiety or stress during this major transitional phase in one’s life (Dayan et al. 2006). Pregnant women worry about the upcoming labour and anticipated pain, also referred to as fear of childbirth (Sjögren 1997), or they may be concerned about the health of the child they are carrying or the physical changes they experience (Huizink et al. 2004). High levels of these anxieties can have adverse health effects on the mother (e.g. Nicholson et al. 2006), and also on the child she is carrying. High pregnancy anxiety levels have been associated with preterm birth and low birth weight (for a review, see Dunkel Schetter and Tanner 2012), and a range of adverse childhood outcomes, including negative emotionality (Gutteling et al. 2005; Huizink et al. 2002), attention deficit hyperactivity disorder (van den Bergh et al. 2005) and developmental delays (Huizink et al. 2003), as well as changes in brain grey matter volume (Buss et al. 2010).

These adverse outcomes associated with pregnancy anxiety indicate that adequate assessment of pregnancy anxiety is important to be able to identify women who have particularly high levels of anxiety during the pregnancy period. This will facilitate prevention and intervention efforts to reduce anxiety during pregnancy, with potentially long-term beneficial effects on the child (Glover 2014). Valid assessment tools to determine the level of pregnancy anxiety are therefore required. The Pregnancy-Related Anxiety Questionnaire–Revised (PRAQ-R), a 10-item shortened version of the PRAQ, has been psychometrically tested (Huizink et al. 2004) and seems to be a robust predictor of birth-related and childhood outcomes, independent of general anxiety measures (e.g. Huizink et al. 2002, 2003; Reck et al. 2013). Moreover, it has been shown in previous studies that pregnancy anxiety assessed with the PRAQ-R reflects a specific construct that can be differentiated from general anxiety for the most part (Huizink et al. 2004, 2014), although the two do influence each other over time during pregnancy (Huizink et al. 2014). Other researchers have shown that pregnancy-specific anxieties are even better predictors of adverse birth and child outcomes than general anxieties (Dunkel Schetter and Tanner 2012; Reck et al. 2013).

Because of its limited number of items in the PRAQ-R, it is a feasible instrument to include not only in scientific studies of pregnant women and their offspring but also in clinical practice. It is therefore a widely used instrument (e.g. Darwiche et al. 2014; Kleinveld et al. 2006; Reck et al. 2013; Vollebregt et al. 2008). The clear disadvantage of this instrument, however, is that it is developed for first-time mothers specifically. Hence, one of the 10 items (i.e. “I am anxious about the delivery, because I have never experienced one before”) is not applicable for the use in women who experienced labour before. Therefore, Westerneng et al. (2015) recently tested the factorial invariance of the total PRAQ-R scale and subscales across nulliparous and parous women. First, they tested the factorial invariance for the 10-item scale, including the ambiguous item for both nulliparous and parous women, and showed that this leads to a non-invariant factor structure in these groups, implying that the questionnaire score cannot be easily compared between nulliparous and parous women. As a second step, they excluded the ambiguous item from the questionnaire data in both groups and ran the same analyses, finding an invariant structure. Thus, their results suggest that when a pregnant study sample consists of nulliparous and parous women, a nine-item PRAQ-R can be used if one wants to combine or compare scores on pregnancy anxiety. However, removing this particular item that is not relevant for parous women altogether can be considered to weaken at least the “Fear of giving birth” factor, which is then only based on two items instead of the original three items. Hence, we considered that a more elegant approach to modifying the PRAQ-R for all women—regardless of their parity—would be to rephrase the item into simply, “I am anxious about the delivery”.

Therefore, we set out to examine whether rephrasing the item “I am anxious about the delivery, because I have never experienced one before”, into the simply shortened version for all pregnant women, i.e. “I am anxious about the delivery”, would yield factorial invariance across nulliparous and parous women. In addition, we examined the extent to which scalar invariance was present to test whether the same constructs are measured on a similar scale (Meredith and Teresi 2006). If this shows to be true, the new modified version of the 10-item PRAQ-R, labelled as PRAQ-R2, could be used and its scores easily compared or combined for data analytic purposes across women of different parities.

## Method

### Participants

The study population was drawn from the ongoing FinnBrain Birth Cohort Study (www.finnbrain.fi), based in Southwestern Finland. The sample initially consisted of the first 1152 women, who were recruited when attending their first trimester ultrasound at gestational week (gwk) 12, and who filled in questionnaires at gwk 24 and gwk 34. Of these women, eight were dropped out because of missing data on parity. The final sample for the current analyses was thus 1144 (608 nulliparous and 536 parous) women. Recruitment of new pregnant women is ongoing and the overall aim is to recruit a total of 4000–5000 families. Thus, the current study sample is a subset of a larger anticipated group of participants.

Women were considered eligible to participate in the study if they had a verified pregnancy, had sufficient knowledge of Finnish or Swedish (the official languages of Finland) to fill in the study questionnaires, and gave written informed consent. In case a severe foetal malformation was revealed during ultrasound, the family was considered non-eligible for the study. Also, miscarriage prior to gwk 25 or stillbirth resulted in non-eligibility for the study.

### Procedure

Recruitment was based on a personal contact by a research nurse. Parent(s) participating in the study gave written informed consent also on behalf of the child. The participants were informed that they could discontinue at any time without having to give an explanation. The Ethical Committee of the Southwestern Finland Hospital District approved of the study.

The research questionnaires covering a wide spectrum of issues related to the well-being of the families were either mailed to the participants or could be filled in online, according to the personal choice of the subjects. The time points of assessment during pregnancy were gestational weeks 14, 24 and 34, and birth. Data was gathered on, for example, the socioeconomic status (SES), smoking and alcohol consumption habits, mental and general health, relationship satisfaction and maternal-foetal attachment. The Pregnancy-Related Anxiety Questionnaire (PRAQ-R and the modified new item) was included at gwk 24 and 34.

### Measures

*Background characteristics* of the participants included maternal age, parity, relationship status, educational level and monthly income. Of the background variables, age was a discrete variable and marital status was dichotomized into single and non-single. Education was trichotomized as follows: low (primary education with/without secondary, e.g. high school or vocational education), middle (college education) and high (university education). Monthly income was categorized into four classes: ≤1000 €, 1001–2000 €, 2001–3000 € and >3000 €. The women included in the study were relatively young so that many were still studying, and thus, they had a relatively low income. Hence, education was considered a better proxy for SES.

*Pregnancy-specific anxiety* was assessed with Finnish and Swedish translations of the 10-item self-report Pregnancy-Related Anxiety Questionnaire–Revised (PRAQ-R; Huizink et al. 2004), a shortened version of the 34-item PRAQ (van den Bergh 1990). Scores on each item ranged from 1 (definitely not true) to 5 (definitely true). The items of the PRAQ-R can be ordered into three subscales. The first subscale, *Fear of giving birth*, consists of three items such as “I am worried about the pain of contractions and the pain during delivery”. The second subscale, *Worries about bearing a physically or mentally handicapped child*, consists of four items, including “I sometimes think that our child will be in poor health or will be prone to illnesses”. The third subscale, *Concern about own appearance*, consists of three items, such as “I am worried about my enormous weight gain”.

For PRAQ, total and factor sum scores were calculated. PRAQ-R items were 2–11 (total sum), and factor sums were F1 (*Fear of giving birth*) sum (items 2, 6 and 8), F2 (*Worries about bearing a physically or mentally handicapped child*) sum (items 4, 9, 10 and 11) and F3 (*Concern about own appearance*) sum (items 3, 5 and 7). For PRAQ-R2, new total sum scores and new F1 sum scores were also calculated, in which item 8 was replaced by the modified item 1 (“I am anxious about the delivery”). See Appendix for all items. In all sum scores, missing values were replaced by factor means (mean value of subject’s responses to other items in the same factor).

### Statistical analyses

Analyses were performed using the Statistical Package of Social Sciences version 20.0 for Windows (SPSS Inc., Chicago, IL) and Mplus (Muthén and Muthén 1998) version 6.0. Descriptive information between the groups was calculated. For reliability analysis, Cronbach’s alpha was used. Mean values with SDs were calculated for all PRAQ items and sum scores. Because, for both nulliparous and parous women, most of the items and total and factor PRAQ sums were skewed (skewness of more than two times the standard error), statistical significances of differences between nulliparous and parous women were evaluated using the Mann–Whitney *U* test, and statistical significances of changes during the study were evaluated using Wilcoxon Signed Ranks test, separately for nulliparous and parous women. Correlations between the PRAQ-R2 items of week 24 and 34 were calculated. Confirmatory factor analyses (CFAs) and measurement invariance were examined on PRAQ-R2 items. First, CFAs on the three factors of the PRAQ-R2 were performed on nulliparous and parous women separately, and then together. The fit of the model was evaluated using different fit indices: *χ*^2^, comparative fit index (CFI), Tucker-Lewis index (TLI), and root-mean-square error of approximation (RMSEA). Because the *χ*^2^ statistic is sensitive to sample size, the fit of the model was found acceptable above 0.90 and good above 0.95 for CFI and TFI (Hu and Bentler 1999) and was found acceptable below 0.08 and good below 0.06 for RMSEA (Hu and Bentler 1999).

Next, measurement invariance across groups of nulliparous and parous women for the PRAQ-R2 scores was examined by testing the factorial invariance (Meredith 1993). One by one, invariance of factor loadings and intercepts were tested. Decrease in model fit was used to compare the models. First, a model was constructed in which factor loadings, intercepts and residual variances of the same indicator variables were all allowed to differ between nulliparous and parous women (model 0). Second, this model was compared to a model in which only factor loadings were constrained to be equal, but residuals and intercepts were allowed to differ between women (model 1). When model 0 showed a better fit than model 1, it would be concluded that the questionnaire measures different constructs between nulliparous and parous women, and thus factorial invariance would be rejected. If model 1 did not fit significantly worse than model 0, metric invariance was considered to be supported, indicating that the questionnaire measures similar constructs between women. Last, a model was estimated in which factor loadings and intercepts were held constrained, but residuals were allowed to differ (model 2). If model 2 fit the data equally well as model 1, scalar invariance was supported, indicating that the same constructs are measured on a similar scale (Meredith and Teresi 2006).

The decrease in model fit was tested using *χ*^2^ difference statistic. This, however, could in large samples lead to statistically significant *χ*^2^ differences when differences are non-substantial or trivial. Therefore, fit decreases were also tested using three other criteria (Koomen et al. 2012): the root deterioration per restriction (RDR; Browne and Du Toit 1992; Dudgeon 2004), the expected cross-validation index (ECVI) difference (Browne and Du Toit 1992; Oort 2009) and the CFI difference (Oort 2009). To calculate the RDR and the ECVI difference with 90 % confidence intervals (CIs), the computer program NIESEM (Dudgeon 2003) was used. If the *χ*^2^ difference was not significant, this indicated that removing a parameter or parameters did not significantly decrease model fit. When the RDR was below 0.08 and the 90 % CI around the ECVI included zero, the models were considered to be equal (Oort 2009). Finally, if the difference between CFI was larger than 0.02, models were considered to be significantly different (Cheung and Rensvold 2002). In cases where factorial invariance did not hold, modification indices were used to indicate which items were non-invariant.

## Results

### Descriptive statistics

Participants’ age ranged between 18.19 and 42.53 at the 24th week of gestation (mean age 30.60, SD = 4.43). Nulliparous women were somewhat younger than parous women (mean age = 29.40, SD = 4.42 for nulliparous; mean age = 31.95, SD = 4.04 for parous women; F (1, 1141) = 102.13, *p* < 0.01). Nulliparous women had a higher mean income than parous women, but the groups were similar in terms of marital status and education (Table 1).

**Table 1:** Mean ages and percentages of marital status, education and income according to parity (*t* test and *χ* ^2^ tests)

		Nulliparous	Parous	*p*
Age	Mean (SD)	29.2 (4.4)	31.8 (4.0)	<0.001
	Range	18–41	20–42	
Marital status (%)	Non-single	98	99	0.643
	Single	2	1	
Education (%)	Low	37	38	0.853
	Middle	34	33	
	High	29	29	
Income (%)	≤1000 €	17	29	<0.001
	1001–2000 €	56	45	
	2001–3000 €	24	21	
	>3000 €	3	5	

In general, nulliparous women tended to have higher PRAQ-R and PRAQ-R2 scores than parous women. The biggest difference between the groups could be observed in item 8, as expected. Item values and factor sum scores in factors 2 and 3 tended to increase during the study, but in factor 3, they tended to decrease. PRAQ-R2 correlations between weeks 24 and 34 were moderately strong, ranging from *r* = 0.47, *p* < 0.01, to *r* = 0.72, *p* < 0.01. For correlations across week 24 and week 34 items, see Table 2.

**Table 2:** Mean (SD) values of PRAQ-R2 items and total and factor sums, and statistical significances of differences between nulliparous and parous women (Mann–Whitney *U* test) and of changes during the study (Wilcoxon Signed Ranks test)

PRAQ items	H24			H34			*p*_CHANGE_	
	Nulliparous	Parous	*p*	Nulliparous	Parous	*p*	Nulliparous	Parous
1	2.5 (1.0)	2.4 (1.1)	0.037	2.6 (1.0)	2.5 (1.1)	0.530	0.005	<0.001
2	2.7 (1.1)	2.5 (1.1)	<0.001	2.9 (1.1)	2.7 (1.1)	0.011	<0.001	<0.001
3	2.7 (1.1)	2.3 (1.1)	<0.001	2.5 (1.1)	2.3 (1.0)	<0.001	<0.001	0.273
4	2.1 (0.9)	1.9 (0.9)	0.030	2.3 (0.9)	2.1 (0.9)	0.001	0.002	0.118
5	2.3 (1.0)	2.3 (1.0)	0.426	2.3 (1.0)	2.2 (1.0)	0.214	0.069	0.038
6	1.8 (1.0)	1.5 (0.8)	<0.001	1.9 (1.0)	1.7 (0.9)	<0.001	0.009	<0.001
7	2.7 (1.2)	2.6 (1.2)	0.173	2.5 (1.2)	2.4 (1.1)	0.052	<0.001	<0.001
8	2.7 (1.3)	1.1 (0.5)	<0.001	2.8 (1.2)	1.2 (0.6)	<0.001	0.860	0.054
9	2.2 (1.0)	2.0 (1.0)	0.016	2.3 (1.0)	2.0 (0.9)	<0.001	0.006	0.880
10	2.2 (1.0)	2.1 (1.0)	0.030	2.3 (1.0)	2.0 (1.0)	<0.001	0.023	0.115
11	2.3 (1.0)	2.1 (1.0)	0.006	2.3 (1.0)	2.1 (1.0)	<0.001	0.093	0.951
NEW total sum	23.5 (6.6)	21.8 (6.3)	<0.001	23.7 (6.6)	21.9 (6.5)	<0.001	0.153	0.487
NEW F1 sum (items 1, 2, 6)	7.0 (2.6)	6.3 (2.4)	<0.001	7.3 (2.5)	6.9 (2.6)	0.002	<0.001	<0.001
F2 sum (items 4, 9–11)	8.8 (3.3)	8.3 (3.3)	0.003	9.1 (3.3)	8.3 (3.3)	<0.001	0.002	0.999
F3 sum (items 3, 5, 7)	7.8 (2.8)	7.2 (2.8)	0.001	7.3 (2.8)	6.8 (2.6)	0.004	<0.001	<0.001

*PRAQ* Pregnancy-Related Anxiety Questionnaire

### Reliability analysis

An overview of all Cronbach’s alphas for PRAQ-R and PRAQ-R2 across the total scale and the subscales, and across nulliparous and parous women, is presented in Table 3. PRAQ-R and PRAQ-R2 Cronbach’s alphas across the total scale were good (above 0.80) and generally comparable. Cronbach’s alphas for the subscale Fear of giving birth were good for the PRAQ-R2: 0.71 and 0.75 for parous women in week 24 and 34 respectively, and 0.79 and 0.75 for nulliparous women in week 24 and 34 respectively. For this subscale in PRAQ-R, Cronbach’s alphas were also good for nulliparous women: 0.78 for week 24 and 0.77 for week 34. However, for the parous women, Cronbach’s alphas were low: 0.40 for week 24 and 0.51 for week 34. Cronbach’s alphas for the subscales Worries about bearing a physically or mentally handicapped child and Concern about one’s own appearance were also good (above 0.77).

**Table 3:** Cronbach’s alphas across the old and new total scale and subscales, separately for nulliparous and parous women

	PRAQ-R		PRAQ-R2	
	Nulliparous	Parous	Nulliparous	Parous
Total scale				
Week 24	0.83	0.80	0.84	0.82
Week 34	0.84	0.83	0.84	0.85
Fear of giving birth				
Week 24	0.78	0.40	0.79	0.71
Week 34	0.77	0.51	0.75	0.75
Worries about bearing a physically or mentally handicapped child				
Week 24	0.77	0.80	0.77	0.80
Week 34	0.80	0.83	0.80	0.83
Concern about one’s own appearance				
Week 24	0.80	0.80	0.80	0.80
Week 34	0.82	0.81	0.82	0.81

*PRAQ-R* Pregnancy-Related Anxiety Questionnaire–Revised, *PRAQ-R2* modified PRAQ-R

### Confirmatory factor analysis

#### Week 24

In week 24 data, CFA based on PRAQ-R2 showed that the three-factor model had an acceptable to good fit in both nulliparous and parous women (*χ*^2^ (32) = 96.88, *p* < 0.01, CFI = 0.98, TLI = 0.97, RMSEA = 0.06 for nulliparous, and *χ*^2^ (32) = 111.19, *p* < 0.01, CFI = 0.97, TLI = 0.96, RMSEA = 0.07 for parous women). Likewise, the three-factor model was fitted on the combined group of nulliparous and parous women, resulting in a good fit (*χ*^2^ (32) =156.81, *p* < 0.01, CFI = 0.98, TLI = 0.97, RMSEA = 0.06). Standardized factor loadings are presented in Table 4.

**Table 4:** Standardized factor loadings of the PRAQ-R2 across the total sample of the three-factor model, for weeks 24 and 34 separately, and correlations between the items across week 24 and 34 for the total sample and parous and nulliparous women separately

Items	Fear of giving birth	Worries about bearing a handicapped child	Concern about own appearance	Correlations		
				Total sample	Parous women	Nulliparous women
1. I am anxious about the delivery	0.85/0.86			0.69**	0.72**	0.67**
2. I am worried about the pain of contractions and the pain during delivery	0.86/0.81			0.66**	0.72**	0.60**
3. I am worried about the fact that I shall not regain my figure after delivery			0.79/0.82	0.61**	0.57**	0.63**
4. I sometimes think that our child will be in poor health or will be prone to illnesses		0.58/0.62		0.51**	0.50**	0.52**
5. I am concerned about my unattractive appearance			0.79/0.79	0.58**	0.57**	0.59**
6. I am worried about not being able to control myself during labour and fear that I will scream	0.47/0.49			0.53**	0.47**	0.55**
7. I am worried about my enormous weight gain			0.70/0.72	0.63**	0.60**	0.66**
9. I am afraid the baby will be mentally handicapped or will suffer from brain damage		0.89/0.89		0.58**	0.56**	0.59**
10. I am afraid our baby will be stillborn, or will die during or immediately after delivery		0.79/0.83		0.61**	0.64**	0.57**
11. I am afraid that our baby will suffer from a physical defect or worry that something will be physically wrong with the baby		0.93/0.94		0.61**	0.60**	0.60**

#### Week 34

The three-factor model was also fitted to the 34-week data. For both nulliparous and parous women, CFA showed an acceptable to good fit to the data (*χ*^2^ (32) = 114.28, *p* < 0.01, CFI = 0.97, TLI = 0.96, RMSEA = 0.07 for nulliparous, and *χ*^2^ (32) = 118.39, *p* < 0.01, CFI = 0.97, TLI = 0.96, RMSEA = 0.07 for parous women). Likewise, the three-factor model was fitted on the combined group of nulliparous and parous women, resulting in a good fit (*χ*^2^ (32) = 190.22, *p* < 0.01, CFI = 0.97, TLI = 0.96, RMSEA = 0.07). For standardized factor loadings, see Table 4.

### Measurement invariance across nulliparous and parous women

#### Week 24

First, a baseline three-factor model was estimated in the week 24 data of PRAQ-R2 without any parous-invariance constraints. Model fit was overall acceptable, *χ*^2^ (65) = 210.58, *p* < 0.01, CFI = 0.97, TLI = 0.96, RMSEA = 0.06. Second, factor loadings were constrained to be equal for nulliparous and parous women. The difference in *χ*^2^ between the first model without constraints and this model with constrained factor loadings showed significant differences, χ^2^_diff_ (7) = 30.84, *p* < 0.01; however, the other three criteria indicated that invariant factor loadings did not result in a significantly worse fit, RDR = 0.077, ECVI_diff_ = 0.01, CI = 0.00–0.03, and ΔCFI = 0.005. In the next step, intercepts were constrained to be equal across groups. Again, χ^2^_diff_ was significant, χ^2^_diff_ (40) = 84.61, *p* < 0.01; however, the other criteria indicated that there were no differences between the model with constraint factor loadings only, and the model with constraint factor loadings and intercepts, RDR = 0.04, ECVI_diff_ = 0.00, CI = −0.02–0.03, and ΔCFI = 0.008.

#### Week 34

In the week 34 data of PRAQ-R2, the same procedure was used. A baseline three-factor model without any parous-invariance constraints was fitted, with an overall acceptable model fit, *χ*^2^ (65) = 232.85, *p* < 0.01, CFI = 0.97, TLI = 0.96, RMSEA = 0.07. Second, factor loadings were constrained to be equal for nulliparous and parous women. There were significant differences in *χ*^2^ between these models, χ^2^_diff_ (7) = 17.11, *p* < 0.01; however, the other three criteria indicated that invariant factor loadings did not result in a significantly worse fit, RDR = 0.05, ECVI_diff_ = 0.00, CI = −0.01–0.02, and ΔCFI = 0.002. Next, intercepts were constrained to be equal across groups. Again, χ^2^_diff_ was significant, χ^2^_diff_ (40) = 59.74, *p* < 0.01, while the other criteria indicated that there were no differences between the models, RDR = 0.03, ECVI_diff_ = −0.02, CI = −0.03–0.00, and ΔCFI = 0.004.

## Discussion

The purpose of the current study was to test whether a slight rephrasing of 1 item of the 10-item Pregnancy-Related Anxiety Questionnaire–Revised (PRAQ-R; Huizink et al. 2004), which is inappropriate for use in parous pregnant women, would result in factorial invariance across nulliparous and parous pregnant women. Rather than deleting this item from the already brief questionnaire for all pregnant women, as Westerneng et al. (2015) recently suggested as a solution for the use of PRAQ-R in a sample of nulliparous and parous pregnant women, we aimed to keep the number of items intact for both groups of women. The advantage of this solution would be that each of the subscales consists of at least three items and hence reflects a more stable and better identifiable factor.

Our results showed that at two time points during pregnancy (i.e. 24 and 34 weeks of gestation), this adaption of one item of the PRAQ-R for parous women leads to metric invariance, indicating that the PRAQ-R2 measures similar constructs among nulliparous and parous women. In addition, our results provided support for scalar invariance across nulliparous and parous women. This implies that the same constructs are measured on a similar scale (Meredith and Teresi 2006). Thus, a slight adaption of one ambiguous item yields a measure that can be easily used, compared or combined in a sample of nulliparous and parous women.

This conclusion based on our data has implications for the use of PRAQ-R in large-scale studies, and for the implementation of the questionnaire in clinical settings or for prevention programs, when an estimate of the level of pregnancy anxiety is required. Our findings support the use of modified PRAQ-R—the PRAQ-R2—in both nulliparous and parous pregnant women. The instrument is brief, valid and feasible, and its predictive abilities have been shown repeatedly, for instance on adverse child behaviours (Gutteling et al. 2005; Huizink et al. 2002; van den Bergh et al. 2005) and on developmental delays (Huizink et al. 2003). Indeed, to underscore the importance of pregnancy anxiety, Glover (2014) recently reported that prenatal anxiety and stress account for 10–15 % of childhood behaviour problems, although in that paper, prenatal anxiety refers to measures of general anxiety mostly, while we argue that pregnancy-specific anxiety is an important factor in predicting child outcomes as well (e.g. Huizink et al. 2014).

The strengths of this study include the large sample of both nulliparous and parous women from the general population in Finland and the repeated assessment of the PRAQ-R in original form with the addition of the modified item. Some limitations have to be taken into account as well, such as that immigrant populations in Finland were excluded when they did not speak Finnish or Swedish sufficiently to be able to understand the questions asked at the assessments. Generalization of the study findings to immigrant populations and to women with particularly high levels of anxiety in clinical settings is therefore not possible.

To conclude, the modified PRAQ-R2 is well suitable for use in pregnant women regardless of parity, as it measures the same constructs repeatedly during pregnancy. Scores of nulliparous and parous pregnant women could be more easily compared and combined if future studies would use this modified wording of one of the items, if they apply the PRAQ-R2 to assess pregnancy-specific anxiety. Better reference scores and materials for all pregnant women would also facilitate screening of pregnant women at particular risk for developing high levels of anxiety (Huizink et al. 2014) and may prove beneficial for child development as well (Glover 2014), thereby enabling the appropriate allocation of prevention and intervention regimes for pregnant women.

## Appendix

The PRAQ-R/R2 form as used in FinnBrain (translated back to English), containing both the old item (8) as well as the new replacing item (1). *: New item applicable for all pregnant women regardless of parity. This item replaces **: Old item only applicable for nulliparous women.

Please circle each answer that applies most accurately to your situation.

Answer categories:

1. Absolutely not relevant
2. Hardly ever relevant
3. Sometimes relevant
4. Reasonably relevant
5. Very relevant

**Table:**

1. I am anxious about the delivery. *	1	2	3	4	5
2. I am worried about the pain of contractions and the pain during delivery.	1	2	3	4	5
3. I am worried about the fact that I shall not regain my figure after delivery.	1	2	3	4	5
4. I sometimes think that our child will be in poor health or will be prone to illnesses.	1	2	3	4	5
5. I am concerned about my unattractive appearance.	1	2	3	4	5
6. I am worried about not being able to control myself during labour and fear that I will scream.	1	2	3	4	5
7. I am worried about my enormous weight gain.	1	2	3	4	5
8. I am anxious about the delivery because I have never experienced one before. **	1	2	3	4	5
9. I am afraid the baby will be mentally handicapped or will suffer from brain damage.	1	2	3	4	5
10. I am afraid our baby will be stillborn, or will die during or immediately after delivery.	1	2	3	4	5
11. I am afraid that our baby will suffer from a physical defect or worry that something will be physically wrong with the baby.	1	2	3	4	5
Total sum scores PRAQ-R:	Items 2–11				
Old subscale PRAQ-R Fear of giving birth:	Items 2, 6, 8				
Total sum scores PRAQ-R2:	Items 1–7, 9–11				
New subscale PRAQ-R2 Fear of giving birth:	Items 1, 2, 6				
Subscale Worries about bearing a handicapped child:	Items 4, 9–11				
Subscale Concern about own appearance:	Items 3, 5, 7				
